# Convolutional neural networks can identify brain interactions involved in decoding spatial auditory attention

**DOI:** 10.1371/journal.pcbi.1012376

**Published:** 2024-08-08

**Authors:** Keyvan Mahjoory, Andreas Bahmer, Molly J. Henry

**Affiliations:** 1 Max Planck Institute for Empirical Aesthetics, Frankfurt am Main, Germany; 2 RheinMain University of Applied Sciences Campus Ruesselsheim, Wiesbaden, Germany; 3 Department of Psychology, Toronto Metropolitan University, Toronto, Ontario, Canada; University of California at Berkeley, UNITED STATES OF AMERICA

## Abstract

Human listeners have the ability to direct their attention to a single speaker in a multi-talker environment. The neural correlates of selective attention can be decoded from a single trial of electroencephalography (EEG) data. In this study, leveraging the source-reconstructed and anatomically-resolved EEG data as inputs, we sought to employ CNN as an interpretable model to uncover task-specific interactions between brain regions, rather than simply to utilize it as a black box decoder. To this end, our CNN model was specifically designed to learn pairwise interaction representations for 10 cortical regions from five-second inputs. By exclusively utilizing these features for decoding, our model was able to attain a median accuracy of 77.56% for within-participant and 65.14% for cross-participant classification. Through ablation analysis together with dissecting the features of the models and applying cluster analysis, we were able to discern the presence of alpha-band-dominated inter-hemisphere interactions, as well as alpha- and beta-band dominant interactions that were either hemisphere-specific or were characterized by a contrasting pattern between the right and left hemispheres. These interactions were more pronounced in parietal and central regions for within-participant decoding, but in parietal, central, and partly frontal regions for cross-participant decoding. These findings demonstrate that our CNN model can effectively utilize features known to be important in auditory attention tasks and suggest that the application of domain knowledge inspired CNNs on source-reconstructed EEG data can offer a novel computational framework for studying task-relevant brain interactions.

## Author summary

In our study, we explored how the brain manages to focus on one speaker among many, a common challenge in noisy environments. Using advanced brainwave (EEG) data analysis, we developed a new method to understand how different parts of the brain communicate during this task. Our technique involves a type of artificial intelligence known as a convolutional neural network (CNN). However, instead of using it as a black box, we designed it to specifically reveal how ten brain areas work together in pairs during selective listening. Remarkably, our approach achieved a high level of accuracy in recognizing where someone’s attention was directed, based on EEG data alone. We discovered that certain patterns of brain activity, especially in regions known for processing sound and spatial awareness, are crucial for focusing attention. This study not only advances our understanding of the brain’s attention mechanisms but also introduces a promising tool for examining complex brain functions, offering insights that could help develop new tools for improving listening in challenging environments.

## Introduction

In a competing-talker situation with noise, a healthy human can focus on a single talker. It has been shown that this focus is reflected in neural activity that more consistently tracks the temporal dynamics of the attended talker’s speech compared to the unattended talker’s speech [1–3]. Auditory selective attention abilities may be weakened or lost as a result of normal aging or hearing impairment [4]. A promising way to potentially counteract selective attention impairment involves the automatic detection of the focus of auditory attention from neural activity and the subsequent amplification of the corresponding audio stream by hearing prostheses [5]. Most studies typically focus on decoding auditory attentional focus using EEG recordings as a non-invasive, portable, and less costly technique as opposed to magnetoencephalography (MEG) or intracranial EEG.

In the past, various approaches have been proposed to decode the brain mechanisms involved in auditory attention from neural time series [1,6–8]. Building on the observation that neural tracking of the amplitude envelope of speech is stronger for attended than unattended material, some studies have attempted to reconstruct the envelope of the attended speech from the EEG signal using linear models [2,9], state-space based models [10,11], canonical correlation analysis [12], and artificial neural networks [13–15]. Other studies have focused on decoding the spatial locus of auditory attention rather than the envelope of the attended speaker [16–20]. Studies focusing on decoding the spatial locus of auditory attention have revealed the importance of activity originating from a frontoparietal network in decoding accuracy [21,22]. In particular, it has been shown that the alpha activity originating from parietal areas is lateralized during selective attention to a location in space, and this lateralization can be leveraged to decode auditory spatial attention [7,23–28]. A recent study has revealed the presence of at least two distinct generators of alpha oscillations over central and parieto-occipital areas during spatial auditory attention [23]. In one study, frontal beta-band activity was shown to be the main predictor of spatial auditory attention [19].

In the last decade, Deep Learning (DL) has emerged as the method of choice for a variety of tasks in computer vision, natural language processing, and audio recognition [29,30]. However, its applications to neural signals have encountered challenges due to certain characteristic properties of neural signals that distinguish them from image or audio data. For example, neural time series possess nonstationary temporal dynamics and spatial patterns occurring in specific frequency bands but typically with a poor signal-to-noise ratio. In addition, EEG recordings contain measurement artifacts like eye movements, heart artifacts, and other unwanted noise sources. These properties substantially change the approach to training artificial neural networks for EEG signal decoding.

Despite the existing challenges, DL has recently demonstrated promise in helping make sense of neurophysiological signals [31,32]. Among several DL techniques, convolutional neural networks (CNNs) have been applied with some success to EEG classification tasks. Indeed, since 2015, CNNs have been the most common architecture type in the majority of EEG studies and their application has been growing steadily [31,33]. The interest in CNNs has been attributed to their innate capability in end-to-end learning and their capacity for extracting temporal and spatial structures in EEG data, as well as their successful applications in computer vision tasks [33,34]. For example, CNNs have been used for decoding auditory attention [19,35], seizure prediction and detection [36,37], and sleep stage classification [38–40].

Some studies have attempted to adapt existing CNN architectures to the task of decoding EEG data specifically, rather than importing them directly from the computer vision applications without modification. These updates in the CNNs architecture have enabled them to learn neurophysiologically interpretable features. For example, Schirrmeister and colleagues introduced a CNN architecture designed that can adapt spatial and temporal filters in the convolutional layers [33]. Along the same lines, Lawhern and colleagues proposed EEGNet, a CNN model adapted to the temporal and spatial properties of EEG data with a relatively small number of parameters to fit, that outperformed other models on four different data sets [34]. In the following years, this type of CNN architecture, built upon learning temporal and spatial features from EEG, has been an active field of research [41–44].

Sensor-level EEG recorded from the scalp, behind the ear, or ear canal, is an obvious choice for real-time applications e.g., the development of neuro-steered hearing aids, because of its portability, low cost, and noninvasive nature. However, EEG sensors capture mixtures of neural activity originating from the entire brain, and not necessarily from the brain area beneath the electrodes [45]. This mixing poses challenges in making interpretations about brain regions based on sensor space EEG. Thus, sensor EEG may be suboptimal in the case that we want to use CNNs to make inferences about the underlying brain regions engaged in auditory decoding.

In this study, we utilized source-reconstructed EEG data as inputs to a novel CNN featuring an interaction-inspired architecture. We attempted to leverage this CNN to pinpoint the cortical regions and their interactions that are pivotal in decoding spatial auditory attention. To our knowledge, this is the first study that attempts to apply CNNs to elucidate the significance of cortical interactions in spatial auditory attention. For this purpose, we used EEG data, recorded from 18 participants attending a speech stream presented to one ear and ignoring speech presented to the other. We reconstructed source time courses for 10 cortical regions comprising the left and the right occipital, temporal, parietal, central, and frontal areas. Time courses of the 10 cortical regions were used as input to our CNN model. In addition, the architecture of our CNN model was specifically designed to enable it to learn internally the interactions between the 10 cortical regions relevant to auditory attention. Our findings demonstrated that the trained CNN model makes use of features that are well-known to be essential for decoding auditory attention.

## Results

### Decoding performance

**Fig 1:**
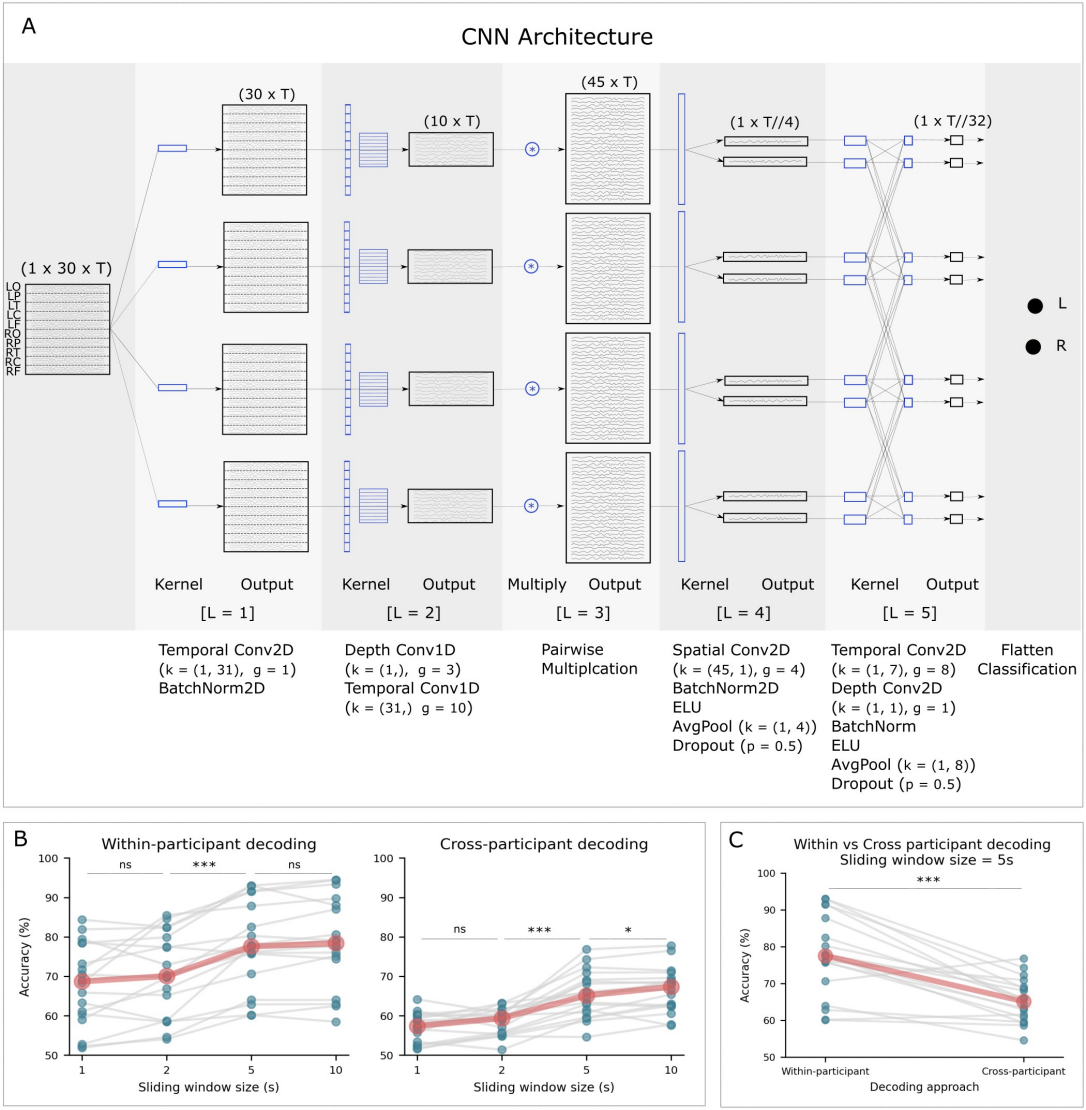
An illustration of our CNN model’s architecture and its performance in decoding spatial auditory attention within and across participants. Panel A: Architecture of the CNN model (detailed architecture in the Materials and methods section). The blue-colored objects represent the CNN kernels applied at each step, and the black objects show the shape of inputs/outputs after each step. For each convolution layer, details including the layer number (L), kernel size (k), and group parameter (g) are specified below each operator. Panel B: Within-participant (left panel) and cross-participant (right panel) auditory attention decoding performance of our CNN model for four different input lengths. A sliding window of 5 s length was determined to be a fair compromise between decoding performance and data length. Blue points show per-participant results averaged over cross-validation folds. Gray lines connect the data points for single participants. Red points represent the median accuracy across participants for each input length. Panel C: Comparing within-participant and cross-participant decoding performance of our CNN model for the same participant and for the input length of 5 seconds.

To decode auditory spatial attention, that is whether the participant was attending to the speech presented to the left or right ear, from source-reconstructed EEG, we employed a CNN model with an architecture that captures interactions between cortical regions and uses them for decoding, as illustrated in Panel A of Fig 1 (see Materials and methods Section for a detailed description of the model architecture). The model input consists of time courses for 30 signals, which correspond to three signals for each of the ten predefined cortical regions, obtained by applying a frequency-band-specific dimensionality reduction technique to enhance activity within the delta-theta, alpha, and beta frequency bands. Input data were generated by moving a sliding window of size T with 50% of overlap over time points. This 50% overlap allows for a more continuous and detailed representation of the EEG data, capturing information that may occur between the windows. It also increases the number of samples available for training the model. Panel B of Fig 1 (and S1 Table) show the decoding accuracy of our CNN model for four different lengths of input data (1 s, 2 s, 5 s, and 10 s), obtained from two training approaches: within-participant (left panel) and cross-participant (right panel). The within-participant decoder for a participant was trained, validated, and tested solely on the data of that participant, using a block-wise four-fold cross validation approach. In contrast, the cross-participant classifier for a participant was trained on data of other participants and then tested on that participant’s data to obtain a participant-independent model which generalizes across participants (see Materials and methods section for further details).

Next, we explored how input length affects decoding accuracy, with input length being the amount of data used to make a single left/right decision. To ensure our results are generalizable and beneficial for neuro-steered hearing aid applications, we focused on shorter decision windows. To statistically test the impact of input length on decoding accuracy, we used a Linear Mixed Effects Model (LMEM), where we specified the length of input data as the independent variable, the decoding accuracy as the dependent variable, and participant as a random effect. Our LMEM analysis, using the Statsmodels library (https://www.statsmodels.org/) implemented in Python, found a significant effect of input length on decoding accuracy (within-participant: p < 0.001, cross-participant: p < 0.001). Furthermore, we found a substantial increase in median decoding accuracy when the input length increased from 2 s to 5 s (Wilcoxon matched-pairs signed rank test, within-participant: W = 2, p < 0.001, cross-participant: W = 1, p < 0.001). Upon extending the input length further from 5 s to 10 s, there was a smaller yet statistically significant enhancement in accuracy (within-participant: W = 39, p = 0.044; cross-participant: W = 37, p = 0.034). We did not observe any significant improvement in decoding performance when input data were increased from 1 s to 2 s for either decoding approach (within-participant: W = 53, p = 0.17; cross-participant: W = 44, p = 0.07). Thus, for the subsequent analyses, we chose the input samples generated by a sliding window of 5 s with 50% overlap, as a good trade-off between the length of input data and the performance of model for the classification task of decoding right from left auditory attention.

Our CNN model achieved a median accuracy of 77.56% for within-participant and 65.14% for cross-participant decoding. Panel C of Fig 1 compares the performance of our CNN model between the within-participant and cross-participant decoding approaches. Unsurprisingly, within-participant classification outperformed cross-participant classification (W = 6, p = 0.001). For the majority of participants (88%), our CNN model performed better when data from the same participant were used for training and testing. Overall, within-participant models tend to perform better than cross-participant models on decoding tasks.

In addition to our CNN model, we conducted a comparative analysis with a baseline model. This baseline model was a binary logistic regression, trained on power values specific to four frequency bands (delta, theta, alpha, and beta) derived from ten distinct cortical regions. The training methodology for this baseline model mirrored that of our CNN approach, encompassing both within-participant and cross-participant decoding. This involved using block-wise cross-validation, as outlined in the Methods section. S1 Fig compares the baseline model’s performance with our CNN, revealing median classification accuracies of 67.56% for within-participant and 61.14% for cross-participant decoding. These figures indicate a median decrease of 10% and 4%, respectively, compared to the CNN model. The Wilcoxon test confirmed that these decreases in performance were statistically significant (within-participant: W = 26, p < 0.01; cross-participant: W = 20, p < 0.01) for both decoding approaches. Furthermore, we compared the decoding performance of our model with the EEGNet [34] and Deep ConvNet [33] models trained on our data. S2 Fig depicts the results. The Wilcoxon test showed the overall superior performance of our model for within-participant (EEGNet: W = 61, p = 0.3; Deep ConvNet: W = 25, p < 0.05) and cross-participant (EEGNet: W = 14, p < 0.01; Deep ConvNet: W = 20, p < 0.05) decoding.

### Brain interactions involved in decoding auditory attention

**Fig 2:**
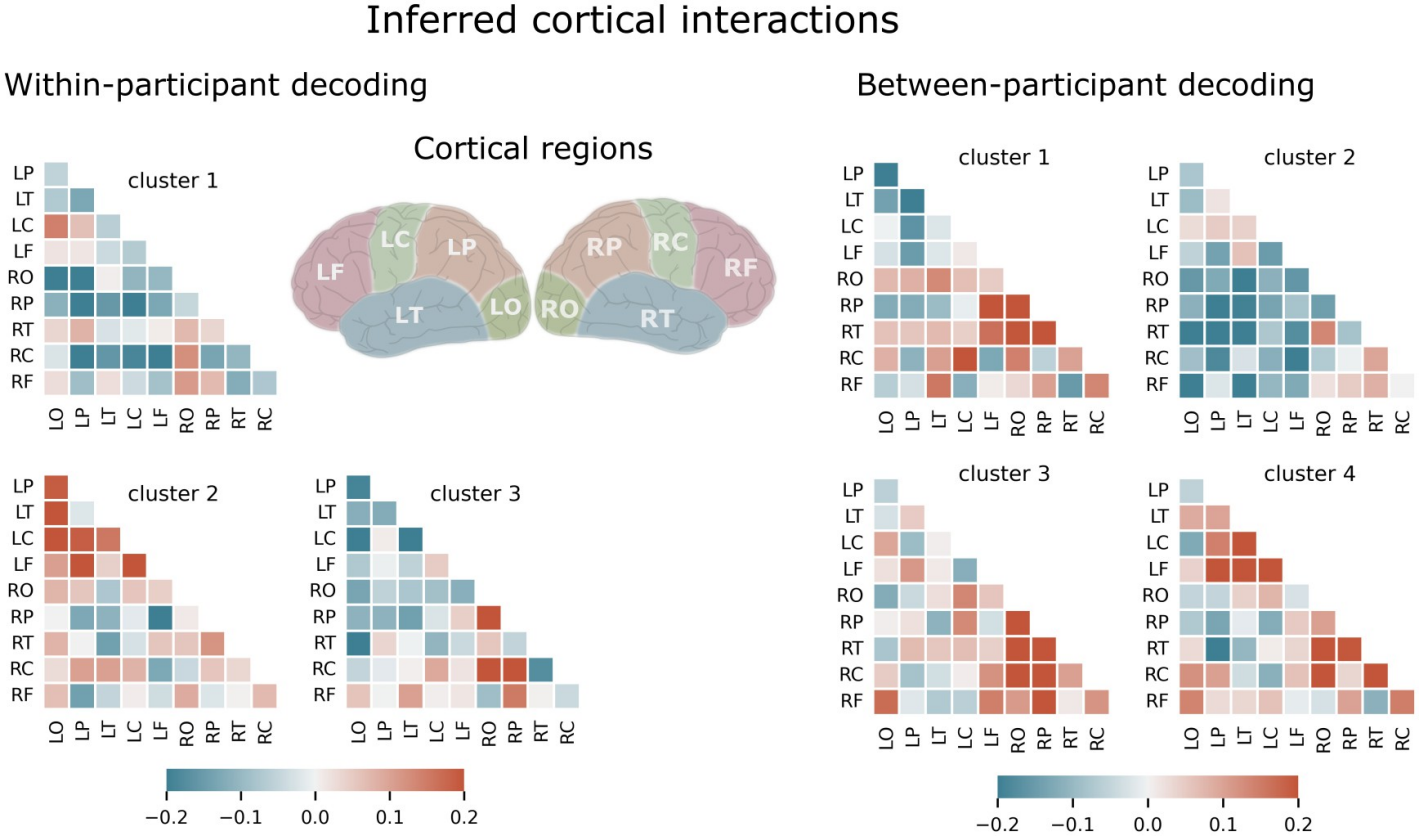
Visualization of the interactions between 10 regions of the brain derived from our CNN model trained using both within-participant (left panels) and cross-participant (right panels) decoding approaches.

The architecture of our CNN model was designed to learn the interactions between ten brain regions of interest (left/right occipital, parietal, temporal, central, and frontal) and then to use only these interactions to decode the locus of auditory attention. This is achieved using spatial filters (45-dimensional vectors) at the fourth convolution layer. These filters serve as a mechanism for the CNN to determine the importance of interactions and indicate which linear combinations of pairwise interactions are utilized by the classifier for the decoding task. We analyzed these spatial filters obtained from the fourth convolution layer. Initially, we focused on CNN models obtained from the within-participant classification approach and extracted spatial filters for all cross-validations and for all participants. We next employed a two-level clustering approach where we applied k-means clustering at the individual level to reduce the data dimensionality to four spatial filters per participant. At the group level, we used hierarchical clustering to group participants with similar spatial filters into clusters. This approach yielded a total of three clusters for the spatial filters obtained from within-participant classification (see Fig 2 left panels).

The first cluster acquired from within-participant decoding comprised a spatial filter that mainly emphasizes the interactions between hemispheres in the occipital, parietal, and central regions (e.g., RO–LP, RP–LP, RP–LC, and RC–LP). These inferred interactions may be related to the lateralization of neural activity during selective auditory attention, as demonstrated by several studies [23,26,27]. In the second cluster, our spatial filter revealed a complex interplay of interactions among areas predominantly within the left hemisphere, notably encompassing the occipital, parietal, and central regions. This pattern suggests the existence of a neural network integrating multiple sensory inputs and cognitive processes. Additionally, these cortical regions demonstrated interaction with frontal areas. Moreover, a notable emphasis on the interaction between the right parietal and left frontal areas was observed. These results are consistent with findings from existing literature on the fronto-parietal network’s pivotal role in spatial auditory attention, suggesting an integration of sensory information (from the auditory and possibly visual systems) with spatial information to effectively guide attention [21,22]. The spatial filter acquired from the third cluster comprised a pattern that assigned negative values for some interactions within the left hemisphere (LO–LC, LO–LP, LT–LC) and positive values for those within the right hemisphere (RO–RP, RO–RC, RP–RC, RP–RF). This spatial filter primarily uses the contrast between the interactions within the right and left hemispheres of the brain.

Analogous to the within-participants analysis, we acquired spatial filters from the cross-participant decoding approach and applied a similar clustering approach at the individual and group levels. This analysis resulted in four clusters of spatial filters (Fig 2, right panels). The first cluster obtained negative values for interactions within the left hemisphere (e.g., LO–LP, LO–LT, LP–LT, and LP–LC interactions) and positive values for those within the right hemisphere (e.g., RO–RP, RO–RT, RO–RP, and RP–RT interactions). This spatial filter utilizes the asymmetry of interactions, the difference between the interactions within the right and left hemispheres, specifically for occipital, parietal, and temporal cortical regions. In contrast, the second cluster highlights between-hemisphere interactions localized mostly to parietal and temporal regions (e.g., RT–LO, RT–LP, RT–LT, RP–LP, RP–LT). The third and fourth clusters, however, showed hemisphere-specific patterns. The spatial filter acquired from the third cluster uses mainly the interactions within the right hemisphere in the occipital, parietal, and temporal areas including e.g., RO–RC, RO–RT, RO–RP, RP–RF, RP–RC, and RP–RT. In the fourth cluster, the spatial filter uses largely the interactions within the left hemisphere between the frontal and all other regions e.g., LP–LF, LT–LF, LC–LF, LT–LC as well as some interactions within the right hemisphere including RT–RO, RT–RP, and RC-RO. These results highlight that the models trained to decode and generalize across participants relied on a combination of within-hemisphere and inter-hemisphere interactions, particularly in the occipital, parietal and temporal areas. This suggests that these cortical regions and their interactions may play a key role in facilitating selective auditory attention across individuals.

### Importance of brain regions and frequency bands for spatial auditory decoding

**Fig 3:**
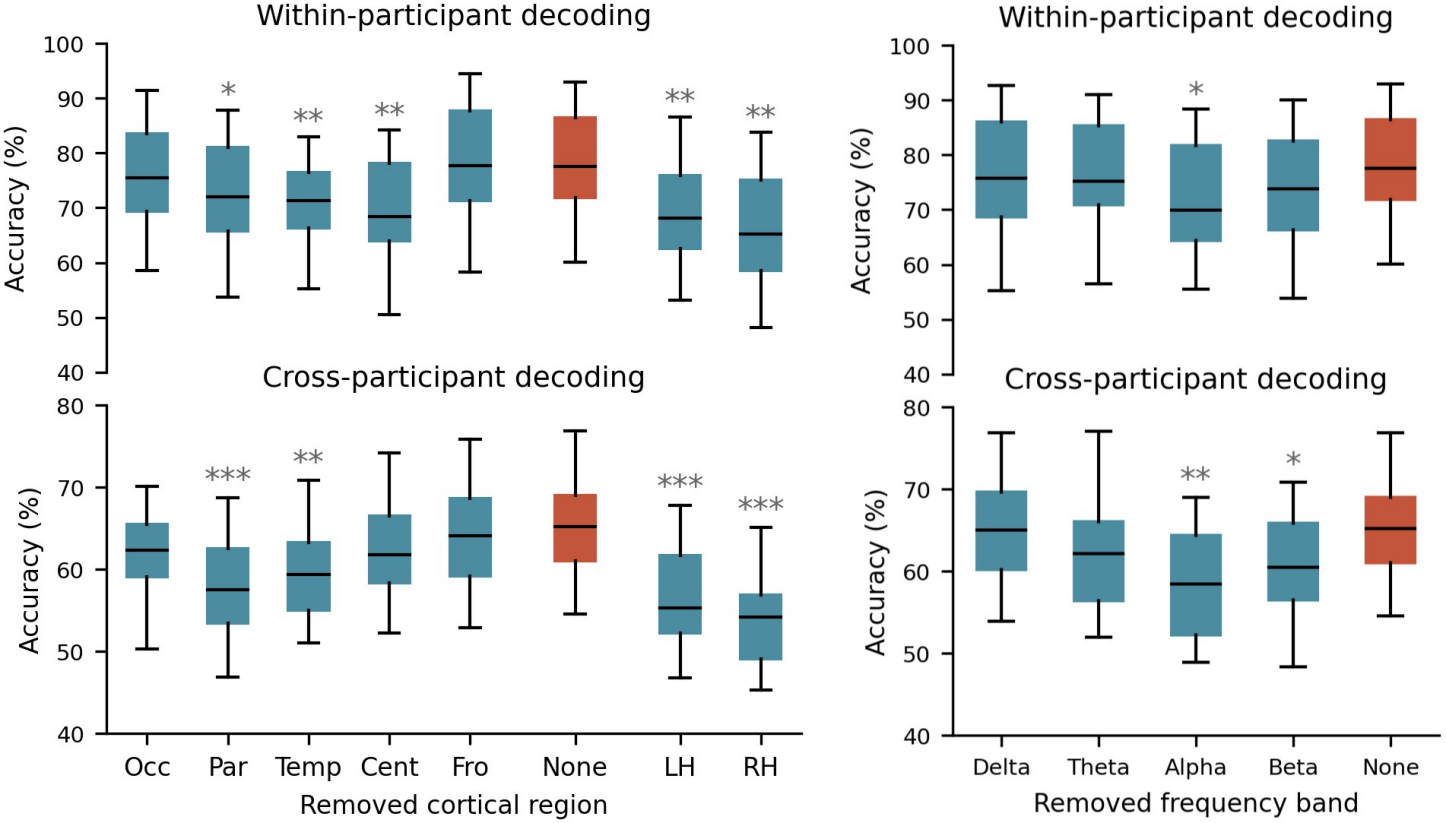
Importance of brain regions and frequency bands for decoding auditory attention. Left panels: performance of our CNN model when particular brain regions were removed from both hemispheres (first five blue boxes from the left), no brain region is excluded (orange box), only left hemisphere regions are removed (the second blue box from right), and only right hemisphere regions are removed (the last blue box). The top panel: within-participant decoding, bottom panel: cross-participant decoding. To remove brain regions from our decoding model, we set the corresponding kernels to zero (Occ: occipital, Par: parietal, Temp: temporal, Cent: central, Fro: frontal, LH: left hemisphere, RH: right hemisphere). Right panels: within-participant (top panel) and cross-participant (bottom panel) auditory attention decoding performance of the CNN model when a certain frequency band is filtered out from the input data. We retested the model (without retraining) by filtering out the delta (2–4 Hz), theta (4–8 Hz), alpha (8–13 Hz), and beta (15–32) frequency bands from test data (blue boxes). The orange box shows the original results.

In order to investigate the significance of the designated cortical areas for the decoding task, the architecture of our model was designed in such a way as to maintain the feature representations associated with ten cortical regions within the initial four convolution layers. This design decision allowed us to conduct a feature ablation analysis (see [34] for further details), where we eliminated the role of a specific brain region in the network by replacing the region-specific elements of the spatial filters (CNN kernels) with zeros. Specifically, we successively removed each of the brain regions (occipital, parietal, temporal, central, and frontal) from the model, and then utilized the resulting brain-region-removed model to decode the test set trials. For this analysis, we chose to remove brain regions from both hemispheres, as the removal of a single brain region from a hemisphere had a negligible impact on decoding performance. In addition to eliminating individual brain regions, we also assessed the performance of the model when all brain regions within either of the left and right hemispheres were removed. The left panels of Fig 3 show the decoding performance of the model when each of the brain regions or hemispheres is removed (blue boxes) as compared to the original model (orange box), for both within-participant (top panel) and cross-participant decoding (bottom panel).

In the within-participant analysis, significant decreases in decoding accuracy were observed following the exclusion of the parietal (median = 72.9%, 4.66% decrease, W = 29, p < 0.01), temporal (median = 71.9%, 5.66% decrease, W = 23, p < 0.01), and central (median = 70%, 7.56% decrease, W = 24, p < 0.01) regions from both hemispheres. In the cross-participant analysis, similar patterns were observed. Exclusion of parietal (median = 58%, 7.14% decrease, W = 7, p < 0.01) and temporal (median = 59.1%, 6.04% decrease, W = 19, p < 0.05) regions significantly decreased decoding performance. Additionally, excluding either full hemisphere resulted in a significant decrease in decoding accuracy for within-participant decoding (LH: median = 68.6%, 8.96% decrease, W = 21, p < 0.001; RH: median = 65.7%, 11.86% decrease, W = 17, p < 0.001) and cross-participant decoding (LH: median = 55.6%, 9.54% decrease, W = 8, p < 0.01; RH: median = 54.5%, 10.64% decrease, W = 1, p < 0.001). All statistical analyses were conducted using paired Wilcoxon tests and corrected for multiple comparisons using False Discovery Rate (FDR) correction for multiple comparisons.

In an attempt to discern the frequency bands employed by our CNN model in decoding auditory attention, we successively filtered out four canonical frequency bands (delta: 2–4 Hz, theta: 4–8 Hz, alpha: 8–13 Hz, and beta: 15–32) from the input data to generate band-removed inputs. Subsequently, we utilized these frequency band-removed data as inputs for the trained models obtained from both within-participant (Fig 3, top-right) and cross-participant (Fig 3, bottom-right) training approaches. The performance of our model on inputs with deleted frequency bands is depicted in blue boxes, while our original findings are presented as a reference in the orange box. It is important to note that, at this stage, we only retested our previously trained models, taking care to prevent any leakage between the test and training sets. The right panels of Fig 3 demonstrate that the elimination of alpha band frequencies from the within-participant decoder (median = 70.4%, 7.52% decrease, W = 25, p < 0.05, FDR corrected), as well as alpha and beta band frequencies from the cross-participant decoder (alpha band: median = 59%, 6.14% decrease, W = 12, p < 0.01; beta band: median = 60.45%, 4.69% decrease, W = 24, p < 0.05, all FDR corrected), significantly decreased the decoder’s accuracy. This finding is consistent with previous research indicating the involvement of alpha [23,46] and beta [19] frequencies in auditory attention.

Consistent with our CNN model, we applied ablation analysis to our baseline model. To explore the impact of cortical regions, we zeroed out weights corresponding to each of the 10 brain regions and retested the ablated model on the test set. The left panel of S2 Fig represents the decoding performance of the ablated baseline model compared to the original baseline model. Elimination of the occipital (median = 59.1%, 2.04% decrease, W = 29, p < 0.05) and central regions (median = 57.77%, 3.37% decrease, W = 32, p < 0.05) significantly reduced the performance of the decoder only for cross-participant decoding. Similar to our CNN model, the exclusion of each full hemisphere from the baseline model significantly reduced the model’s performance for both within-participant and cross-participant decoding (within-participant, LH: median = 56.36%, 11.2% decrease, W = 16, p < 0.01; RH: median = 57.42%, 10.14% decrease, W = 22, p < 0.05; cross-participant, LH: median = 51.24%, 9.9% decrease, W = 7, p < 0.001; RH: median = 54.13%, 7.01% decrease, W = 6, p < 0.001, all FDR corrected). Next, to investigate the impact of frequency bands, we zeroed out weights corresponding to each frequency band and retested the ablated model on the test set. The right panel of S2 Fig depicts the decoding performance of the ablated baseline model compared to the original baseline model for both within-participant and cross-participant approaches. Notably, only the exclusion of alpha frequencies from the original model significantly impacted the performance of the model (median = 57.3%, 3.8% decrease, W = 25, p < 0.05, FDR corrected). Our analysis indicates that the CNN model, in comparison to the baseline, not only achieves higher accuracy but also more effectively utilizes spatial and frequency information from the data.

### Frequency specificity of identified interactions for spatial auditory decoding

**Fig 4:**
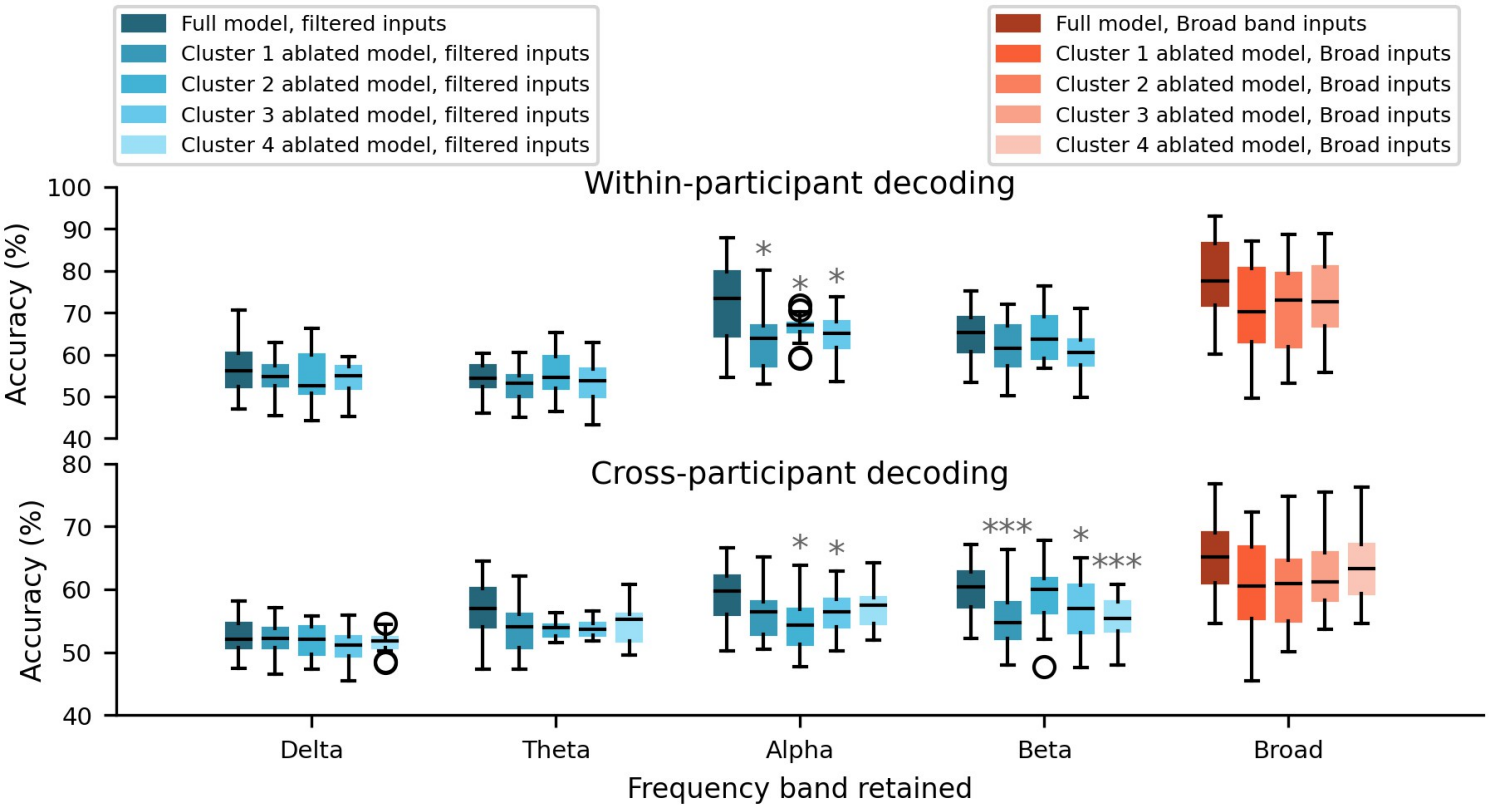
Performance of our convolutional neural network model when data filtered within four frequency bands (delta, theta, alpha, and beta) were used as inputs. Both the original CNN model (dark-colored boxes) and ablated models (light-colored boxes) were tested using within-participant (top panel) and cross-participant (bottom panel) decoding approaches. Results for broad-band (2–32 Hz) inputs are shown in orange boxes. The ablated models were constructed by removing the impact of significant interactions identified by our cluster analysis. Statistical analysis for each frequency band is performed by comparing the performance of ablated models with the full model.

Results of the analyses conducted in the previous subsections revealed significant roles of alpha and beta band frequencies in decoding accuracy. These analyses also highlighted the importance of interactions, primarily among the occipital, parietal, and central regions, for within-participant decoding, as well as among the parietal and temporal regions for cross-participant classification. In an effort to thoroughly explore the interrelationship between these two findings derived from independent analyses, we sought to determine whether the identified interactions are specific to a particular frequency band. To address this question, we iteratively filtered the data within the delta, theta, alpha, and beta frequency bands and constructed band-specific inputs. Additionally, we conducted a feature ablation analysis on our CNN model in which we eliminated the influence of the interactions identified through our cluster analysis. This was achieved by first Z-transforming the values within each spatial filter relative to the mean and standard deviation of that filter, then converting each Z-value to a p-value using the normal cumulative distribution function and retaining only those values with p < 0.05 (uncorrected). The ablated model was created by zeroing out the elements determined to be significant interactions in the fourth convolution layer. Finally, we tested both the original and ablated models with the band-filtered inputs without re-training. Fig 4 illustrates the decoding accuracy of the original model (dark-colored boxes) versus ablated models (light-colored boxes). This comparison is made for four filtered inputs (blue boxes) and for broad-band inputs (orange boxes) using within-participant and cross-participant decoding approaches. Here, an ablated model was created by removing the significant interactions of the corresponding cluster from the original model.

In the within-participant decoding analysis, removing significant interactions identified by clusters 1, 2, and 3 significantly reduced decoding accuracy, but only when the model was fed with the alpha-band-retained inputs (full model: median = 73.4%; cluster 1 ablated model: median = 63.87%, 9.53% decrease, W = 24, p < 0.05; cluster 2 ablated model: median = 67.07%, 6.34% decrease, W = 35, p < 0.05, cluster 3 ablated model: median = 65.01%, 8.39% decrease, W = 33, p < 0.05; all FDR corrected). The accuracy decrease was less pronounced and not statistically significant when the model was fed with the unfiltered data or data filtered to delta, theta, and beta bands. In the cross-participant decoding approach, we observed a large decrease in performance when using alpha-specific inputs with clusters 2 and 3 removed (full model: median = 59.66%; cluster 2 ablated model: median = 54.27%, 5.4% decrease, W = 24, p < 0.05; cluster 3 ablated model: median = 56.45%, 3.21% decrease, W = 31, p < 0.05; all FDR corrected), or beta-band inputs with clusters 1, 3, and 4 eliminated from the model (full model: median = 60.36%; cluster 1 ablated model: median = 54.68%, 5.68% decrease, W = 18, p < 0.001; cluster 3 ablated model: median = 57.0%, 3.36% decrease, W = 32, p < 0.05; cluster 4 ablated model: median = 55.33%, 5.03% decrease, W = 6, p < 0.001; all FDR corrected). However, when the model was fed with delta, theta specific, or broad-band inputs, the omission of interactions from the model did not result in a significant decrease. Our results show that the cortical interactions identified by our cluster analysis are dominated by alpha and beta band frequencies.

## Discussion

In this study, we utilized source-reconstructed and anatomically-resolved EEG data as inputs for a novel CNN topology that we propose as an interpretable and transparent model for enhancing our understanding of the significance of cortical interactions in decoding auditory spatial attention. Our CNN model was specifically designed to learn pairwise interaction representations for 10 cortical regions including the left and the right occipital, parietal, temporal, central, and frontal areas. Using only these features for decoding, our model achieved median accuracy of 77.56% for within-participant classification and 65.14% for cross-participant.

Upon analyzing the spatial filters learned by the CNN model, we uncovered the presence of three main types of interactions utilized by the model for the decoding task: inter-hemisphere interactions, hemisphere-specific interactions, and the contrast between interactions within the right and left hemispheres. The inter-hemisphere interactions were localized in the occipital, parietal, and central regions for the within-participant decoding approach, and in the parietal, temporal, and frontal regions for the cross-participant decoding approach. Further ablation analysis revealed that these interactions were significantly dominated by alpha frequencies in both decoding approaches. Hemisphere-specific interactions were localized only in the left hemisphere for within-participant decoding, but in the right hemisphere or a combination of both hemispheres for cross-participant decoding. Our analysis also demonstrated that these interactions were largely dominated by alpha frequencies, with some contribution from beta frequencies. Finally, the pattern of interactions encapsulating the difference between interactions within the right and left hemispheres primarily utilized interactions between parietal and central regions for within-participant decoding, and interactions between the parietal and temporal regions for cross-participant decoding. Ablation analysis showed that both alpha and beta frequencies contributed to these interactions.

These findings, obtained from our analysis approach, reflect the results of previous EEG studies demonstrating the presence of at least two distinct generators of alpha oscillations over central and parieto-occipital regions [23,26,27] and lateralization of parietal alpha activity as the main indicator of spatial attention [7,23–25,28]. In addition, our results are further supported by a recent study that applied a CNN model to sensor-space EEG data and uncovered the involvement of beta-band activations, primarily in the frontal and temporal regions, in the decoding of spatial auditory attention [19]. Our confirmatory results demonstrate the utility of our analysis approach, which employs a CNN architecture built based on the characteristics of anatomically resolved EEG data, in utilizing features known to be important in auditory attention. As a result, this strategy can be utilized as a tool to deduce additional characteristics of brain functioning related to auditory attention. The proposed model was not designed for direct real-time application; instead, its results are aimed at guiding the development of EEG-based methodologies optimized for real-time use. Our contribution to this approach is to propose an architecture that is informed by domain-specific knowledge and maintains operational transparency. This transparency is crucial, as it allows users to understand the underlying decision-making processes of the model. At the same time, our model retains the flexibility to unravel complex, non-linear relationships within the data. This balanced approach suggests our method could be a valuable asset in the ongoing evolution of computational neuroscience.

Although our findings highlight the crucial role of alpha and beta frequencies in decoding spatial attention, we observed a minimal effect of lower frequencies, such as delta and theta bands. This concurs with recent research reporting the trivial impact of low frequencies on auditory attention [19]. However, it is possible that this may also be due to the architecture of the CNN model, which may not be sufficiently deep to capture longer structures in the data. In the context of deep neural networks applied to computer vision, it is well-established that initial convolution layers capture simple structures, while deeper layers build upon one another and learn to encode more abstract structures in the data [47]. However, implementing a deeper CNN would lead to an increase in the number of parameters and, in our case, overfitting due to the relatively small number of trials per participant and a small overall number of participants. Other factors that can affect the frequency of inputs include the size of convolution kernels and the use of pooling layers. In our study, in order to supply the interaction operator with a broad range of frequencies, we set the size of convolution kernels to be half of the sampling frequency, allowing the convolution to capture all frequencies above 2 Hz and included average pooling layers only after the interaction layer. Alternative network architectures that may be more effective in capturing longer temporal structures like delta and theta frequencies in the data include those designed for language modeling such as recurrent neural networks or convolution-based approaches like WaveNet [29,48].

In the present study, in line with several studies which have attempted to adapt existing CNN architectures to the properties of EEG data rather than importing them directly from the computer vision applications [33,34,41,43], we sought to utilize CNNs not merely as a high-performing "black box" decoder, but rather as an interpretable tool for deriving task-specific interactions between brain regions. To achieve this, we first transformed the input data into an interpretable structure by source projecting the EEG data and obtaining time series for individual cortical regions of interest. Additionally, we integrated the shape of the input data within the architecture of the model by maintaining the anatomical information in the initial four convolution layers. This way, the model was pushed to leverage the existing prior domain knowledge and subsequently utilize it for the decoding task. This input and architecture integrated approach allowed us extract task-specific interactions from trained models.

Although the median accuracy of our decoding pipeline was 77.56% for within-participant and 65.14% for cross-participant training approaches, it varied among participants for both within-participant and cross-participant training approaches. For example, as shown in Fig 1, for some participants the decoding performance was better than 90%, whereas for two participants it was around chance level. This discrepancy may be related to the relatively low number of trials per participant for the within-participant approach and the small number of participants for cross-participant generalization. However, we were unable to determine why certain individuals had low performance in our decoding process. To properly account for this inter-individual heterogeneity, particularly for real-time applications like hearing aids, future research may use more data, employ data augmentation techniques, utilize more flexible CNN architectures, and/or include behavioral data.

In this study, we employed a 64-channel EEG for source-reconstruction to learn and subsequently infer brain interactions from trained models. However, high-density EEG systems or MEG data, which offer higher spatial resolution, may yield more precise source localization. This increased precision could enhance the interpretation of neural interactions and, consequently, the accuracy of the model. Our approach is adaptable to other EEG/MEG experiments involving various decoding tasks by applying minor adjustments to the architecture of our CNN model.

Comparing the performance of our CNN model with a logistic regression baseline model and two other well-established models, EEGNet and Deep ConvNet, reveals a significant outperformance of our model in both within- and cross-participant decoding. The ablation analysis suggests that the enhanced performance of our CNN model may be attributed, at least in part, to its utilization of a broader spectrum of frequency bands and more extensive cortical region data. This indicates the CNN’s potential for more effective extraction of relevant neural information from inputs and efficient utilization of these features, suggesting its capability in handling the complexities of auditory spatial attention decoding. The core of our study was to present a brain-interactions-inspired CNN architecture with a transparent feature extraction design. Future studies may explore other architectures to adapt the model to brain dynamics or interactions, such as incorporating attention layers or graph convolutional neural networks, among other potential approaches [44,49–53].

Although entrainment-based mechanisms have also been proposed to discern the direction of auditory attention [54,55], our research focused exclusively on neural interactions and did not investigate entrainment patterns relative to stimuli. However, a promising direction for future research could involve developing a CNN model that integrates both stimulus information, such as speech envelopes, and EEG signals to decode auditory attention. This model could be designed with interpretability in mind, aiming to extract valuable insights into entrainment mechanisms.

In conclusion, we presented a CNN that takes in time series data from 10 cortical regions and learns to extract the relevant interactions between them for decoding auditory spatial attention. Our interpretable model design is based on the properties of EEG time series data. By interpreting the trained models, we found that the network was able to identify known important interactions in spatial auditory attention and that alpha and beta frequencies played a key role in its performance. Overall, our CNN approach provides a promising approach for exploring and understanding neural dynamics and their interactions involved in decoding tasks.

## Materials and methods

### Dataset

We used publicly available EEG data (https://zenodo.org/record/1199011#.XDyExfzgpyA) recorded from 18 participants with normal hearing. EEG was recorded from 64 channels (BioSemi ActiveTwo system) with sampling rate of 512 Hz. Two additional electrodes were also placed on the mastoids as physiological reference signals [56].

During the experiment, sixty 50-s long trials were recorded, during which participants listened to two simultaneously presented speech streams (one on the left and one on the right) and were cued to listen to one and ignore the other. Virtual auditory environments (VAEs) were simulated using the room acoustic modeling software Odeon. The binaural VAEs were reproduced in a soundproof, electrically-shielded listening booth with ER-2 insert earphones (Etymotic Research). To spatially separate speech signals presented via earphones, the speech signals were convolved with non-individualized head-related impulse responses for azimuth angles of ±60° and an elevation angle of 0°. The order of presentation of different virtual auditory environments (anechoic, mild reverberation, and high reverberation) was independently randomized across trials for each participant. Moreover, the position of the target speaker relative to that of the listener (±60°) as well as the gender of the target speaker were randomized across blocks for each participant. The presentation order of the stories was also randomized across participants. For further details about the data and experiment see [56]. In this study, we employed stratified sampling to ensure an equal proportion of different conditions across the training, validation, and test sets.

### EEG preprocessing

We used the MNE-Python package [57,58] to analyze the data. EEG time series were band-pass filtered using a zero-phase forward filter with range 2–32 Hz and down-sampled to 64 Hz. EEG data were then epoched based on the provided trigger information, and we discarded trials where only a single speaker was presented. Next, the time series of each channel was visually inspected and excessively noisy channels/time segments were manually rejected. Data were re-referenced to the common average of channels. Infomax-based independent component analysis [59] was applied to identify and remove artifactual components from the EEG recordings (average: 4.7, std: 2.5). Retained components were back-projected to the sensor space.

### EEG source analysis

To reconstruct time courses of the cortical sources generating the scalp EEG activity, we carried out source reconstruction, which requires solving forward and inverse models. The forward model describes the physical process of neuronal current propagation from the dipolar sources constrained to cortical regions to the EEG channels. To compute the forward model, we used a standard template anatomy based on FSAverage included in the Freesurfer package (https://surfer.nmr.mgh.harvard.edu), and extracted the inner skull, outer skull, and outer scalp surfaces, each comprising 1280 nodes. These three surfaces were used to obtain a three-layer head model (conductivity of the layers was set to MNE-Python defaults: 0.3, 0.006, 0.3). The source space was defined as a dipole grid on the white matter surface down-sampled to 5124 source points using the topology of a recursively subdivided icosahedron (“ico-4” option). Finally, the forward model was computed, using the boundary element method (BEM) as implemented in the MNE-Python package, between sources constrained to the cortical surface and 64 EEG electrodes projected to the scalp surface.

Three-dimensional dipolar sources were reconstructed under free-orientation using the minimum norm estimate method [60] with depth weighting of 0.8 to compensate for the depth bias towards the superficial sources [61]. The cortical surface was parceled into 10 coarse regions-of-interest (Fig 2, middle panel) including right and left occipital (RO, LO), right and left parietal (RP and LP), right and left central (RC, LC), right and left frontal (RF, LF), right and left temporal (RT, LT) regions according the Desikan-Killiany atlas [62] to obtain time courses for each region.

To reduce the number of time series per ROI, we performed dimensionality reduction using the Generalized Eigenvalue Decomposition broadband (GEDb) approach [63]. GEDb seeks components that enhance signal to noise ratio in a certain frequency band of interest by solving a generalized eigenvalue decomposition problem where the covariance of band-pass filtered data is specified as the signal covariance and the covariance of the broadband data as the noise covariance matrix (or reference covariance matrix) [63,64]. GEDb was carried out on each ROI across time courses of sources within the ROI, at delta-theta (2–8 Hz), alpha (8–13 Hz), and beta (15–32 Hz) frequency ranges, separately. Only the first component of GEDb was retained yielding three time series (corresponding to three frequency bands) per ROI and 30 time series per participant.

Next, by applying a sliding window of size T and with an overlap of 50% over time points, we generated the input samples of size 30 x T for the classifier. We tested four values for T (1 s, 2 s, 5 s, and 10 s).

Our goal in applying source reconstruction and GEDb analysis was to provide anatomically-resolved and SNR-enhanced data for our CNN model and ultimately be able to interpret our results in a neurophysiologically meaningful way.

### Convolutional neural networks

A convolutional neural network (CNN) is a type of artificial neural network that consists of a series of convolutional layers and nonlinear activation functions. These layers employ kernels or filters of a specified size, which slide over the data to extract local features. When applied to EEG data, these convolution kernels can extract either temporal or spatial features, depending on their shape. For instance, a row kernel can be used to learn temporal features, while a column kernel can be utilized to extract spatial information hidden in the raw data. Convolutional layers are often followed by pooling layers, which down-sample the feature space but retain important features. Activation functions introduce nonlinearity to the model. The CNN is optimized by minimizing a loss function using an optimization algorithm, which estimates the optimal parameters (kernels) for the model.

Our proposed CNN for decoding auditory attention is shown in Panel A of Fig 1. The architecture of this model is partly inspired by an existing CNN called EEGNet (Lawhern et al., 2018) [34]; however, it has been modified to learn interactions between 10 cortical regions and utilize only those features for the decoding task. The input matrices to the model have dimensions of 30 x T. Here, 30 represents signals obtained from 10 distinct brain regions. Each of these regions contributes three signals, each specifically enhanced for one of the three targeted frequency bands: delta-theta, alpha, and beta. The T represents the number of time points captured within each analysis’s sliding window. We used GEDb to accentuate the activity within these frequency bands of interest.

In the initial layer of our model (L = 1), we employed a two-dimensional convolution with four two-dimensional kernels of size 31 (k = (1, 31)), yielding four feature maps of dimensions 30 x T. The length of the temporal kernel was set to 31, corresponding to half the sampling rate, thereby enabling the model to capture frequencies within the range of 2–32 Hz. This convolution layer was then succeeded by batch normalization along the time points [65].

In the second layer, we first utilized a one-dimensional convolution layer (k = (1,), g = 3). This operation is equivalent to applying a distinct convolution operator to each of the 10 cortical regions, processing three signals per region. It results in a single output signal for each region. Through this operation, the model learns a linear combination of the three signals for each cortical region. We then implemented a one-dimensional temporal convolution with 10 groups of convolution kernels, each of size 31 (k = (31,)). This operation further refines the information extracted by the previous layer by enabling the model to learn more complex patterns within each signal. It is particularly beneficial when the signals contain a mixture of frequencies or when the patterns of interest are not temporally synchronized across signals.

Next, we performed element-wise multiplication between all pairs of the 10 time series, resulting in four matrices with dimensions 45 x T that represent the interaction time series. This operation forces the model to learn the interactions between the 10 time series and use these features for the decoding task.

In the following convolution layer, we used a two-dimensional spatial convolution of size (45, 1) to learn spatial filters from the interaction time series that determine which linear combination of interaction time series is most important for the decoding task. We then applied batch normalization and the exponential linear unit (ELU) activation function [66]. To reduce the number of time points, we employed an average pooling layer of size (1, 4). The average pooling operation reduces the sampling frequency of the data to 16 Hz. The kernels utilized as spatial filters here were also constrained to maintain a maximum norm of one. After that, a dropout with a probability value of 0.5 was applied [67]. The dropout operation helps to prevent overfitting by randomly dropping out a portion of the activations in the layer during training, effectively forcing the model to learn more robust features.

The subsequent layers of our CNN model were designed similar to the architecture of EEGNet [34]. Specifically, we employed a separable convolution, which consists of a depth-wise convolution followed by pointwise convolutions [68]. Unlike standard convolution, which performs both spatial and temporal computation in a single step, separable convolution divides the computation into two distinct stages: the depth-wise convolution applies a single convolutional filter to each input channel, while the pointwise convolution combines the outputs of the depth-wise convolution via a linear operation. When applied to EEG data, this technique allows the model to learn feature maps in the time domain and subsequently combine these maps in an effective manner. In addition, separable convolution also has the advantage of reducing the number of parameters to be learned.

In the final layer, the classification layer, the inclusion of a fully connected layer was omitted in order to decrease the number of parameters and incorporate features indicating interactions across brain regions directly into the classifier. To do that, the features were flattened and passed to a two-unit softmax classifier [34,69]. The model was implemented using Pytorch (https://pytorch.org).

### Model training and evaluation

To train, evaluate, and test our model, we used an approach similar to the procedure described by Lawhern and colleagues [34], and performed within-participant and cross-participant analyses to assess the decoding of auditory attention.

For the within-participant decoder, we employed a block-wise, four-fold cross validation approach. Each participant’s data was divided into four blocks. Of these, two blocks were randomly designated as the training set, one block as the validation set, and the final block as the test set. For the cross-participant decoder, we utilized each participant’s full dataset as the test set in five distinct iterations. In each iteration, the data from four randomly selected participants were employed as the validation set, while the data from the remaining 13 participants formed the training set. As this procedure was systematically repeated five times for each of the 18 participants, a total of 90 different folds for cross-validation were generated. This approach ensures that each participant’s data is comprehensively tested and validated in various training and validation contexts.

In both the within-participant and cross-participant decoders, we used the training set for training the model and updating its parameters. The validation set was utilized for ongoing evaluation, hyperparameter tuning, and model selection. Finally, the test set was exclusively used for testing the final model to assess and report its performance. The model training involved minimizing the cross-entropy loss between the predicted outputs and the actual labels, using the Adam optimizer [70]. We set the batch size to 32 and omitted the bias term from all convolutional layers. We ran 200 training epochs with validation stopping, saving the model parameters that produced the lowest validation set loss. To evaluate the model, we chose accuracy as our metric, given the nearly balanced distribution of classes among all participants.

### Clustering

After training the CNNs for all cross validations and individuals, we extracted the spatial filters learned in the third convolution layer for all models. We then employed k-means clustering at the individual level followed by a hierarchical clustering at the group level to partition the spatial filters into distinct clusters.

At the individual level, to reduce the data dimensionality, the spatial filters obtained from cross-validations per participant were grouped into four clusters using the k-means algorithm. The k-means algorithm treats each spatial filter as a point in 45-dimensional space and groups data into k mutually exclusive clusters by minimizing the centroid distance of observations within clusters and maximizing the distance between clusters. We used the cosine distance metric. The number of clusters was set to four, consistent with the number of spatial filters in our CNN model. The choice of four clusters for k-means was also matched with the optimal number of k-mean clusters identified by the Elbow curve and the Silhouette methods (see S5 Fig). The k-means algorithm was repeated 10 times with different (randomly determined) centroids and maximally 100 iterations. The solution with the smallest sum of distance values within the clusters was accepted.

At the group level, we employed a hierarchical clustering approach to group participants with similar spatial patterns into subgroups. In a bottom-up manner, this algorithm initially treats each data point as a cluster of its own and then further pairs of clusters are successively merged as one moves up the hierarchy. We used cosine angle as the metric for the linkage computation. Finally, based on our visual inspection of the resulting dendrograms and local maxima of the resulting silhouette values, we chose three clusters for within-participant analysis and four clusters for the cross-participant training approach. For our clustering analyses we used the Scikit learn package (scikit-learn.org).

### Baseline model and comparative models

We have incorporated a baseline model: a binary logistic regression trained on power values specific to four frequency bands (delta, theta, alpha, and beta bands), derived from 10 cortical regions. Structurally, this model ingests a 40-dimensional feature vector (power values for four frequency bands and 10 cortical regions). It then processes these through a singular output neuron with a sigmoid activation function, yielding a value ranging from 0 to 1, indicative of the probability associated with one of the two classes under consideration.

For comparison, we chose EEGNet and Deep ConvNet as established. In the EEGNet model, we set the number of channels to 30, corresponding to the number of time series from 10 brain regions, with each region contributing three time series. Given that we down-sampled the data to 64 Hz, we employed Conv2d filters of size (1, 32) in the first block, with the filter length set to half the sampling rate, as described in the original paper. The rest of the EEGNet architecture remained identical to the original [34]. For the Deep ConvNet, we used the model as described in Fig 1 by Schirrmeister and colleagues [33]. We adhered to the architecture detailed in their publication.

Utilizing binary cross-entropy as our loss function and Gradient Descent for optimization, the model’s training was conducted analogously to our proposed CNN model. The training encompassed both within-participant and cross-participant decoding, leveraging block-wise cross-validation, as detailed above in the training and evaluation subsection.

### Statistical analysis

We employed the Wilcoxon signed-rank test, a non-parametric statistical test known for its robustness in handling non-normally distributed data, to compare the models’ performance, using the scipy.stats.wilcoxon function from the SciPy library. For each comparison, we calculated the W statistic, which represent the sum of the ranks for the observations where the first condition exceeds the second. To address the issue of multiple comparisons in our comparative analysis, we applied False Discovery Rate (FDR) correction, utilizing the scipy.stats.false_discovery_control function.

## Supporting information

S1 Fig Accuracy comparison between the CNN model (red) and the baseline logistic regression model (blue) for decoding auditory spatial attention. The box plots depict accuracy distributions for within-participant and cross-participant decoding. The central mark represents the median accuracy, with the edges of the box indicating the 25th and 75th percentiles. The asterisks indicate the level of statistical significance in performance differences as determined by the Wilcoxon test (**p < 0.01). The results show a statistically significant superior performance of the CNN model over the baseline for both within-participant and cross-participant decoding.
(TIFF)

S2 Fig Comparison of accuracy between our CNN model (red) and other models (blue), including EEGNet and Deep ConvNet, for decoding auditory spatial attention. The results demonstrate statistically significant superior performance of our CNN model over EEGNet and Deep ConvNet in both within-participant and cross-participant decoding.
(TIFF)

S3 Fig Significance of brain regions and frequency bands in decoding auditory attention using a logistic regression baseline model. Left panels show the baseline model’s performance with the exclusion of specific brain regions from both hemispheres (first five blue boxes), no exclusion (orange box), removal of only left hemisphere regions (second blue box from the right), and only right hemisphere regions (last blue box). Top panel: within-participant decoding; bottom panel: cross-participant decoding. Brain regions were excluded by setting the corresponding kernels to zero (Occ: occipital, Par: parietal, Temp: temporal, Cent: central, Fro: frontal, LH: left hemisphere, RH: right hemisphere). Right panels depict the performance of a CNN model for within-participant (top) and cross-participant (bottom) decoding, when specific frequency bands are omitted from the input data. Performance was tested without retraining after filtering out delta (2–4 Hz), theta (4–8 Hz), alpha (8–13 Hz), and beta (15–32 Hz) frequency bands from the test data (blue boxes). The original results are shown in the orange box. The asterisks indicate the level of statistical significance in performance differences as determined by the Wilcoxon test (*, **, *** for p < 0.05, p < 0.01, p < 0.001, respectively). Here, each ablated model (blue box) was compared with the corresponding original model (orange box).
(TIFF)

S4 Fig Performance comparisons of within-participant (WP) and cross-participant (CP) decoders when tested on the same participants vs new participants. The figure illustrates the decrease in classification accuracy of within-participant decoders when tested on new, unseen participant data, highlighting their limited generalizability (W = 3, p < 0.001). Conversely, the cross-participant decoders, trained on data from 13 participants and tested on different data sets from the same participants, show a significant increase in classification accuracy (W = 11, p < 0.001).
(TIFF)

S5 Fig Elbow curve of sum of squares error (SSE) and silhouette score used to determine the optimal number of clusters (k) in k-means clustering analysis. (TIFF)

S1 Table Mean and Standard Deviation of Accuracy Scores for the CNN Model for Various Input Data Length. (PDF)
