# Supplementary figures and images for: Convolutional neural networks can identify brain interactions involved in decoding spatial auditory attention
Source: PLoS Comput Biol. 2024 Aug 8;20(8):e1012376. doi: 10.1371/journal.pcbi.1012376 (PMC11335149; doi:10.1371/journal.pcbi.1012376)

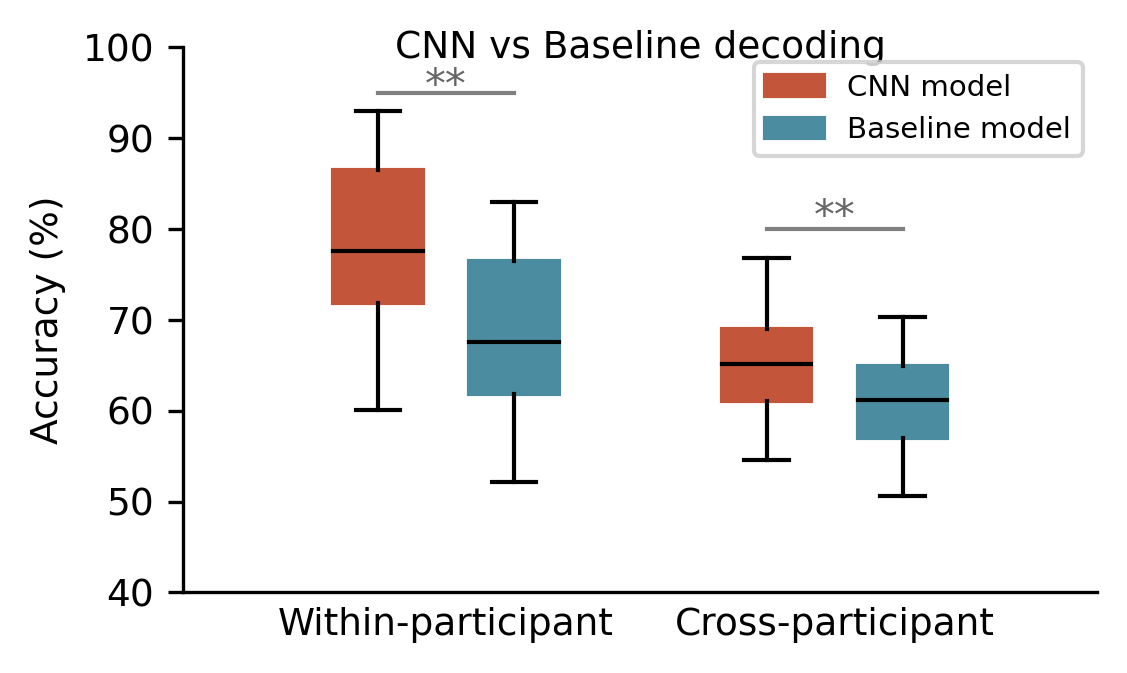

Supplement: S1 Fig — The box plots depict accuracy distributions for within-participant and cross-participant decoding. The central mark represents the median accuracy, with the edges of the box indicating the 25th and 75th percentiles. The asterisks indicate the level of statistical significance in performance differences as determined by the Wilcoxon test (**p < 0.01). The results show a statistically significant superior performance of the CNN model over the baseline for both within-participant and cross-participant decoding. (TIFF) [file pcbi.1012376.s001.tiff]

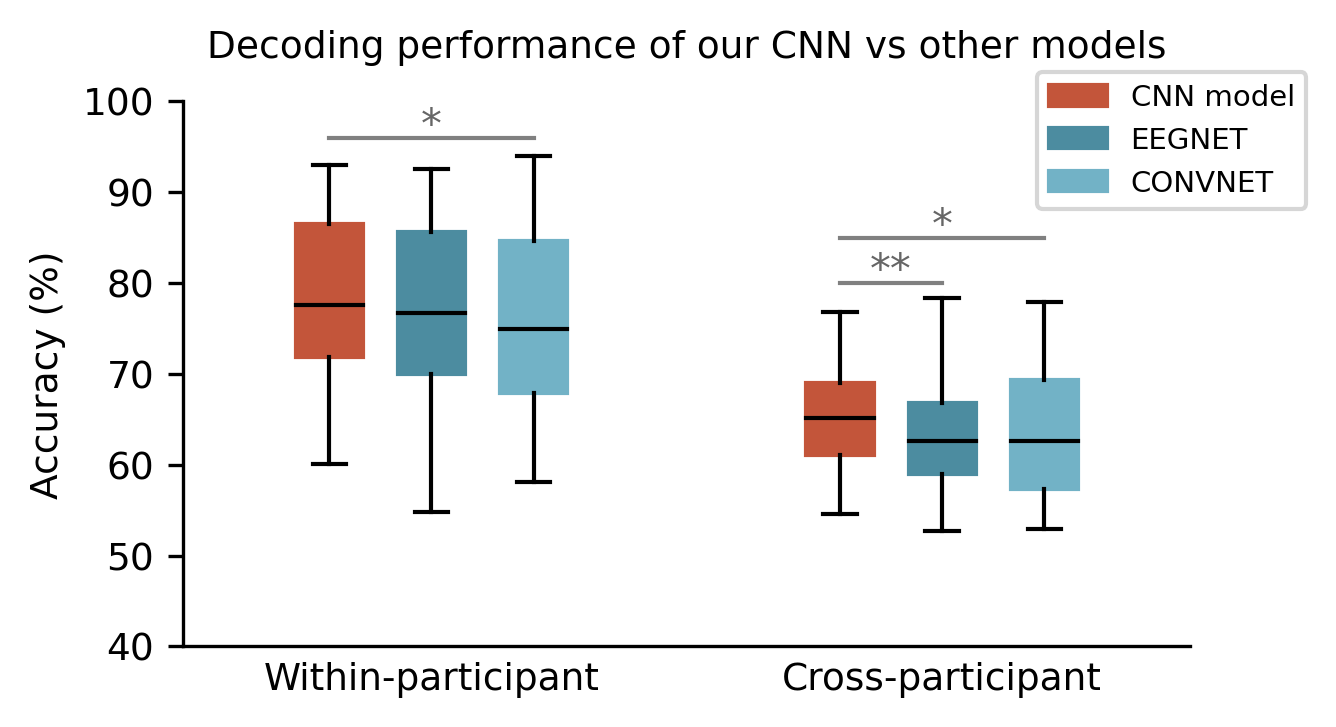

Supplement: S2 Fig — The results demonstrate statistically significant superior performance of our CNN model over EEGNet and Deep ConvNet in both within-participant and cross-participant decoding. (TIFF) [file pcbi.1012376.s002.tiff]

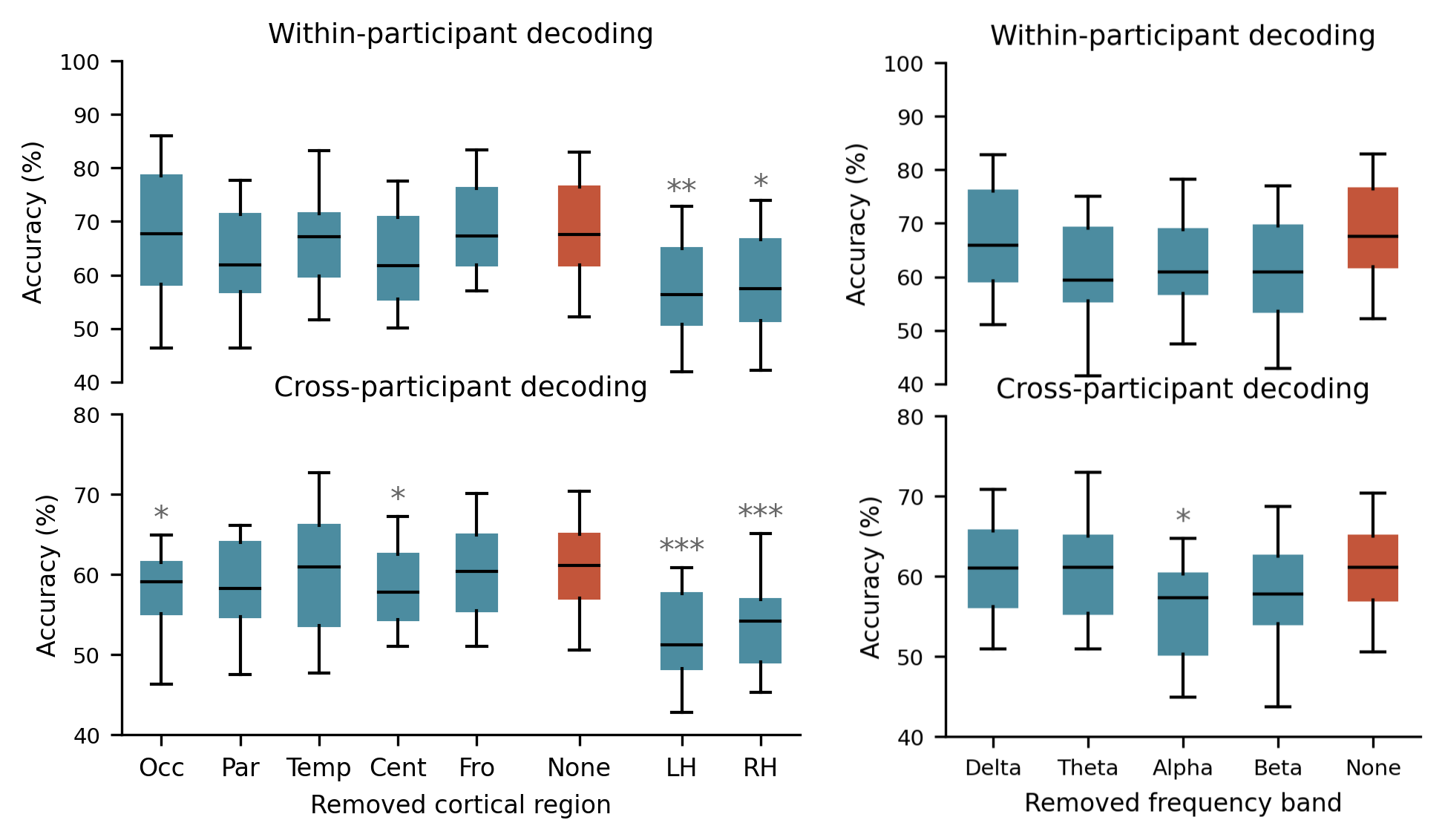

Supplement: S3 Fig — Left panels show the baseline model’s performance with the exclusion of specific brain regions from both hemispheres (first five blue boxes), no exclusion (orange box), removal of only left hemisphere regions (second blue box from the right), and only right hemisphere regions (last blue box). Top panel: within-participant decoding; bottom panel: cross-participant decoding. Brain regions were excluded by setting the corresponding kernels to zero (Occ: occipital, Par: parietal, Temp: temporal, Cent: central, Fro: frontal, LH: left hemisphere, RH: right hemisphere). Right panels depict the performance of a CNN model for within-participant (top) and cross-participant (bottom) decoding, when specific frequency bands are omitted from the input data. Performance was tested without retraining after filtering out delta (2–4 Hz), theta (4–8 Hz), alpha (8–13 Hz), and beta (15–32 Hz) frequency bands from the test data (blue boxes). The original results are shown in the orange box. The asterisks indicate the level of statistical significance in performance differences as determined by the Wilcoxon test (*, **, *** for p < 0.05, p < 0.01, p < 0.001, respectively). Here, each ablated model (blue box) was compared with the corresponding original model (orange box). (TIFF) [file pcbi.1012376.s003.tiff]

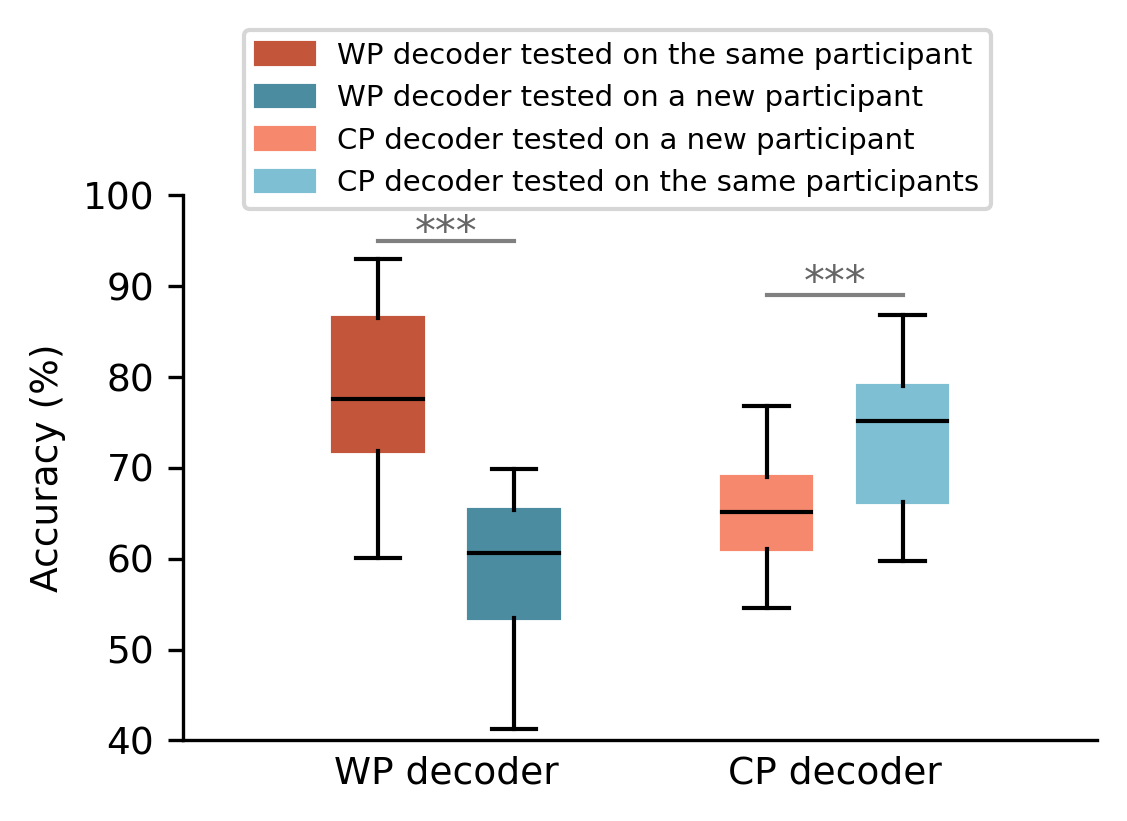

Supplement: S4 Fig — The figure illustrates the decrease in classification accuracy of within-participant decoders when tested on new, unseen participant data, highlighting their limited generalizability (W = 3, p < 0.001). Conversely, the cross-participant decoders, trained on data from 13 participants and tested on different data sets from the same participants, show a significant increase in classification accuracy (W = 11, p < 0.001). (TIFF) [file pcbi.1012376.s004.tiff]

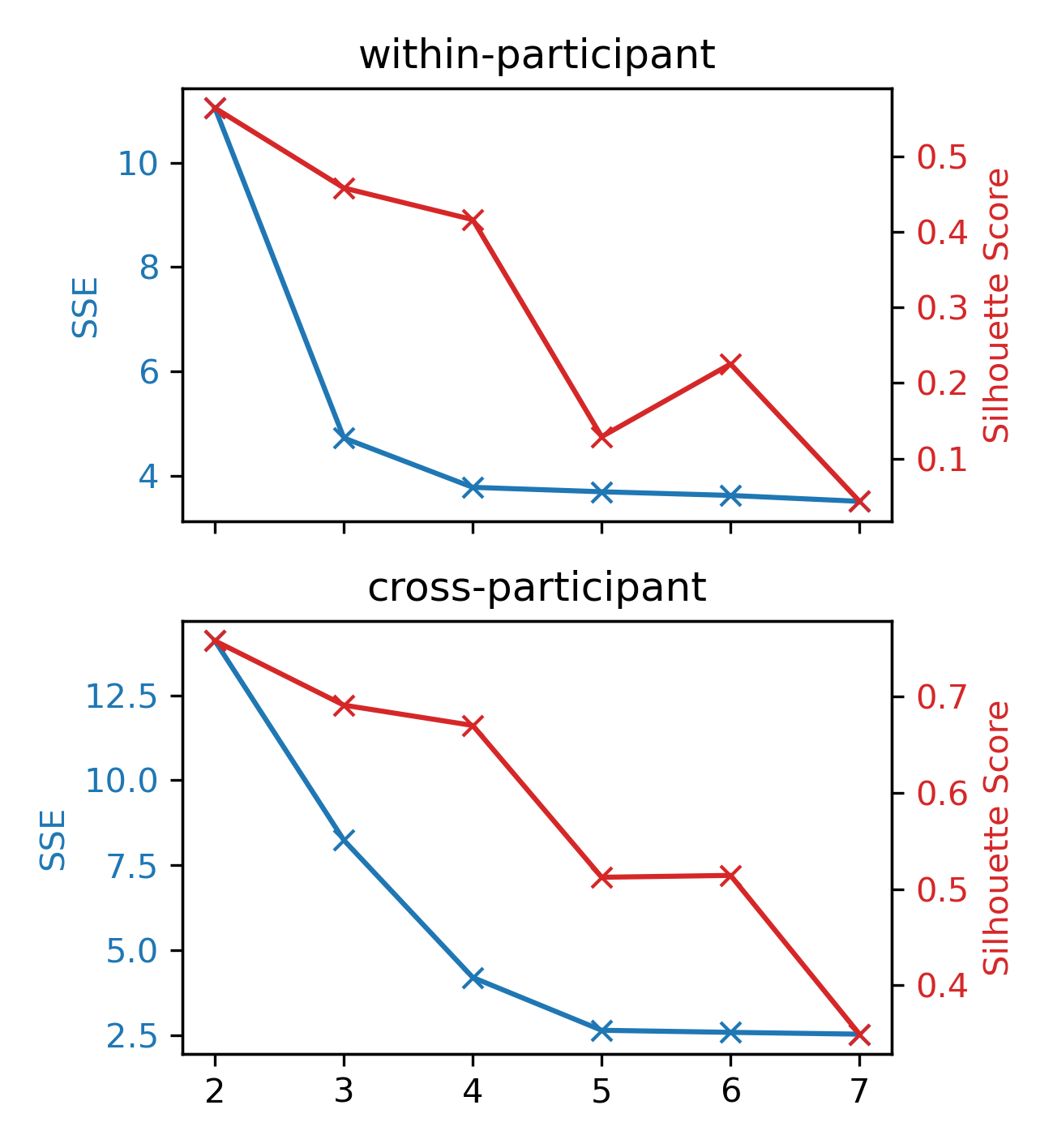

Supplement: S5 Fig — (TIFF) [file pcbi.1012376.s005.tiff]
