# Supplementary material for: Convolutional neural networks can identify brain interactions involved in decoding spatial auditory attention
Source: PLoS Comput Biol. 2024 Aug 8;20(8):e1012376. doi: 10.1371/journal.pcbi.1012376 (PMC11335149; doi:10.1371/journal.pcbi.1012376)
Supplement: S1 Table — (PDF) [file pcbi.1012376.s006.pdf]

Input length (s)	Within-participant decoder		Cross-participant decoder	
	mean ACC (%)	std ACC (%)	mean ACC (%)	std ACC (%)
1	68.69	10.03	57.38	3.6
2	70.04	9.25	59.33	3
5	77.56	10.7	65.14	5.81
10	78.40	11.5	67.1	5.53
